# Effect of eCG on Terminal Follicular Growth and Corpus Luteum Development and Blood Perfusion in Estrous-Synchronized White Lamphun Cattle

**DOI:** 10.3390/ani15060867

**Published:** 2025-03-18

**Authors:** Molarat Sangkate, Punnawut Yama, Atsawadet Suriard, Wichayaporn Butmata, Setthawut Thammakhantha, Noppanit Daoloy, Anukul Taweechaipaisankul, Chih-Jen Lin, Pin-Chi Tang, Tossapol Moonmanee, Jakree Jitjumnong

**Affiliations:** 1 Department of Animal and Aquatic Sciences, Faculty of Agriculture, Chiang Mai University, Chiang Mai 50200, Thailand; molarat.s@cmu.ac.th (M.S.); punnawut.yama@rmutl.ac.th (P.Y.); assawadet_suriard@cmu.ac.th (A.S.); wichayaporn_b@cmu.ac.th (W.B.); setthawut_t@cmu.ac.th (S.T.); noppanit_da@cmu.ac.th (N.D.); tossapol.m@cmu.ac.th (T.M.); 2 Department of Animal Science and Fisheries, Faculty of Agricultural Science and Technology Lanna, Phitsanulok 65000, Thailand; 3 Department of Biology, Faculty of Science, Chulalongkorn University, Bangkok 10330, Thailand; anukul.t@chula.ac.th; 4 Centre for Reproductive Health, Institute for Regeneration and Repair (IRR), University of Edinburgh, Edinburgh EH16 4 UU, UK; chih-jen.lin@ed.ac.uk; 5 Department of Animal Science, College of Agriculture and Natural Resources, National Chung Hsing University, Taichung 40227, Taiwan; pctang@dragon.nchu.edu.tw; 6 The iEGG and Animal Biotechnology Center, National Chung Hsing University, Taichung 40227, Taiwan; 7 Functional Feed Innovation Center, Faculty of Agriculture, Chiang Mai University, Chiang Mai 50200, Thailand

**Keywords:** blood perfusion, corpus luteum development, Doppler analysis, equine chorionic gonadotropin (eCG), follicular dynamics

## Abstract

Equine chorionic gonadotropin (eCG) has been shown to improve reproductive performance in cattle by stimulating terminal follicular growth. This study was designed to assess the effects of 400 IU of eCG on TFG and its consequences for luteal development in indigenous cows undergoing ovulation synchronization. Specifically, we investigated the follicular and luteal dynamics following eCG treatment using structural markers (follicular and luteal diameters and luteal area) and functional markers (P4 profiles and luteal blood perfusion) to evaluate CL development. A total of twelve cows were assigned into two groups: an untreated control group and an eCG-treated group (*n* = 6 in each). Ultrasonography was used to scan the ovaries and monitor the development of TFG and ovulation from day 0 to ovulation. The diameter in TFG was measured daily, and the terminal follicle (TF) was identified as the preovulatory follicle throughout the study period. Ovulation was defined by the disappearance of the TF. Following ovulation, the progression of CL development in both groups was observed on days 5–6, and the mid-stage CL was identified between days 9 and 12 post-ovulation. The results indicated a faster increase in the terminal follicular diameter (TFD). From day 4 to ovulation, the eCG-treated group showed a significantly greater terminal follicular growth rate (TFGR) compared to that in the untreated control group. This enhanced follicular growth in the eCG-treated group was associated with more robust CL blood perfusion and maturation. The Doppler imaging assessments revealed a significant increase in the blood perfusion within the CL, which corresponded with the accelerated TFG. Pearson’s correlation analysis showed strong positive relationships between follicular growth, luteal function, and CL blood perfusion, underscoring the interconnectivity of these processes. In conclusion, our results highlighted the key role that eCG administration has in TFG and CL development and function in White Lamphun cattle.

## Simple Summary

This study evaluated the effects of eCG treatment on the terminal follicular growth (TFG) and corpus luteum (CL) development in multiparous White Lamphun cows. Twelve cows were randomly assigned into either an untreated control group or an eCG-treated group. Both groups followed the same ovulation synchronization protocol, which included a 7-day CIDR, PGF2α, and GnRH protocol, but the eCG-treated group also received an eCG dose at day 4 to stimulate TFG. The results demonstrated that eCG treatment significantly accelerated TFG and enhanced CL development, including through a greater CL size and luteal area. Doppler imaging revealed increased blood perfusion in the CL, while higher plasma P4 levels, measured through ELISA, were observed in the eCG group, particularly during the mid-stage CL phase. Correlation analyses indicated strong associations between TFG and CL parameters, as well as a significant relationship between the Doppler imaging, P4 levels, and CL area. These findings suggest that faster TFG leads to more robust luteal growth and higher P4 production, highlighting the beneficial effects of eCG on ovarian function and its potential to improve the reproductive performance in White Lamphun cows. However, further studies with larger sample sizes and the use of fixed-timed artificial insemination (FTAI) are needed to confirm these results.

## 1. Introduction

White Lamphun cattle (*Bos indicus*), a native breed of northern Thailand, are highly valued due to their adaptability to tropical climates, high fertility, and low nutritional requirements. Hormonal treatments for synchronizing estrus and ovulation are considered the most effective approach to improving the estrus and ovulation synchronization efficiency in beef cattle herds [1,2]. The success of ovulation synchronization programs depends on the coordination between the ovulatory follicle and the corpus luteum (CL), which is closely linked to follicular dynamics and the functions of both the CL and the ovulatory follicle following the synchronization protocol. However, many aspects of these protocols remain underexplored, particularly regarding supplementary hormonal treatments [3], ovarian follicular characteristics [4], and breed differences (*Bos indicus* vs. *Bos taurus*) [5].

In beef cows, the commonly used 7-day Co-Synch protocol for synchronizing ovulation involves the administration of exogenous hormones, including gonadotropin-releasing hormone (GnRH), controlled internal drug release (CIDR), and prostaglandin F2α (PGF2α) [6]. To optimize the efficiency of this approach, a deeper understanding of reproductive physiology—specifically follicular and CL development—is essential for improving reproductive management. Reproductive efficiency in cattle is often compromised by challenges such as poor estrus detection and a high prevalence of anestrus, particularly in tropical breeds [7]. Fixed-time artificial insemination (FTA) procedures facilitate the use of AI by eliminating the need for estrus detection and allow for the precise timing of insemination. However, the success of FTAI depends on the induction of synchronized ovulation in a healthy follicle, which subsequently develops into a functional CL capable of producing adequate progesterone (P4) levels to support pregnancy [8]. In cattle, follicles acquire an ovulatory capacity at diameters of approximately 10 mm (*Bos indicus*) or 12 mm (*Bos taurus*) [9,10]. Terminal follicular growth (TFG) and the size of the ovulatory follicle are critical determinants of oocyte quality, ovulation, and P4 production during the early luteal phase—factors that are vital for successful pregnancy outcomes [11]. Elevated P4 levels during the early stages of pregnancy are particularly important for embryo development and survival [12,13].

Equine chorionic gonadotropin (eCG), a glycoprotein with both follicle-stimulating hormone (FSH)- and luteinizing hormone (LH)-like activity, has been widely used in FTAI protocols to enhance ovarian follicular growth and improve the pregnancy rates in cattle [14,15,16,17]. Its prolonged half-life (approximately 45 h in cows) provides sustained gonadotropic support, making it particularly beneficial for postpartum anestrus cows, which often exhibit an insufficient LH pulse frequency to sustain TFG and ovulation [18,19]. While eCG has been shown to improve the follicular growth and P4 production in *Bos indicus* cattle [14], its effects on TFG, CL formation, and CL blood perfusion in indigenous breeds like White Lamphun cattle remain unexplored.

The CL is a transient but highly vascularized ovarian structure that plays a pivotal role in establishing and maintaining pregnancy. It develops from the ovulated follicle and produces P4, which is essential for regulating uterine function and supporting embryo development [20,21,22]. The developmental competence of bovine oocytes is significantly influenced by the preovulatory follicular environment and the dynamic changes occurring before the LH surge [23]. Developmental competence refers to an oocyte’s ability to mature, be fertilized, and progress to the blastocyst stage. This competence is gradually acquired during the oocyte growth phase, in parallel with follicular development [24]. The blood perfusion of the CL, which can be assessed using Doppler ultrasonography, is closely linked to its functional capacity and P4 production [25]. Enhanced blood perfusion to the CL improves the delivery of nutrients and hormones, thereby supporting its development and function [26,27].

To the best of our knowledge, no studies have yet investigated the effects of adding eCG to the estrous synchronization protocol on TFG and CL development. Based on these observations, we hypothesized that the administration of eCG within the estrous synchronization protocol would enhance TFG, subsequently influencing CL development. Therefore, the main objective of this study was to evaluate the effects of incorporating eCG into the estrous synchronization protocol on the TFG, CL development, and CL function in White Lamphun cattle, with specific assessments of growth rate, blood perfusion, and P4 secretion.

## 2. Materials and Methods

### 2.1. Animal Care and Management

Animal care and use were approved by the Institutional Animal Care and Use Committee (IACUC) of the Faculty of Agriculture, Chiang Mai University (Approval No. RAGIACUC027/2567). A total of twelve anestrous, multiparous, suckled White Lamphun cows (*Bos indicus*), aged 3 to 5 years, weighing 218.25 ± 6.05 kg, and ranging from 30 to 60 days postpartum, were selected for this study. The cows had a body condition score of 5.0 on a scale from 1 to 9 (where 1 = emaciated and 9 = obese) [28]. The experiment was conducted between November and December 2024. Prior to the experiment, the cows were dewormed and vaccinated against hemorrhagic septicemia and foot-and-mouth disease (FMD). All of the animals were housed in an indoor corral at the Beef Cattle facility, located at the Agricultural Innovation Research, Integration, Demonstration, and Training Center (AIRIDTC), the Faculty of Agriculture, Chiang Mai University (Latitude: 18°45′ N/Longitude: 98°55′ E). The cows were fed a diet consisting of silage and a mixed ration, provided ad libitum. Additionally, they received a commercial concentrate supplement (4% of their body weight), which contained 16% crude protein, 13% moisture, 12% crude fiber, and 3% crude fat. Water, salt, and minerals were provided ad libitum throughout the duration of the experiment.

### 2.2. The Experimental Design and Treatment Protocols

**Figure 1:**
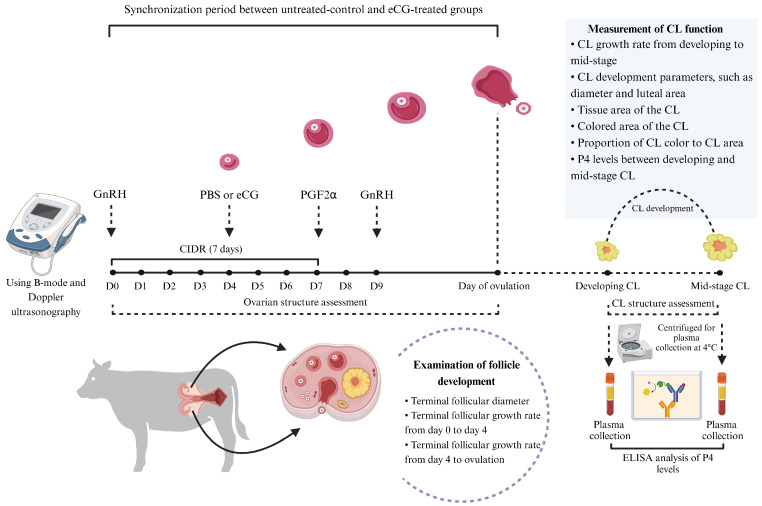
Experimental design and treatment protocols. Twelve multiparous White Lamphun cows (*n* = 12) were randomly assigned into two groups: the untreated control group (*n* = 6) and the eCG-treated group (*n* = 6). On the first day of synchronization (designated as day 0), a CIDR device containing 1.38 g of P4 was inserted into the vagina for 7 days. An intramuscular injection of 10 μg of GnRH was administered as the first injection, followed by a PGF2α injection after the CIDR device’s removal on day 7. On day 9, a second 10 μg GnRH injection was administered. Additionally, the eCG-treated group received 400 IU of eCG on day 4, while the untreated control group received an equivalent volume of PBS.

The experimental cows were randomly assigned into two groups, a phosphate-buffered saline (PBS)-treated group (untreated control; *n* = 6) and an equine chorionic gonadotropin (eCG)-treated group (eCG-treated; *n* = 6), with both groups consisting of White Lamphun cows. On the first day of synchronization (designated as day 0), a controlled internal drug release (CIDR) device (Eazi-Breed CIDR, Zoetis Inc., Auckland, New Zealand) containing 1.38 g of P4 was inserted into the vagina for 7 days (from day 0 to day 7). Additionally, an intramuscular injection of 10 μg of gonadotropin-releasing hormone (GnRH) (Buserelin, Receptal, MSD Animal Health, Upper Hutt, Wellington, New Zealand) was administered as the first injection. Following the removal of the CIDR device on day 7, a 500 μg injection of prostaglandin F2α (PGF2α) (Cloprostenol, Estrumate, MSD Animal Health, Upper Hutt, Wellington, New Zealand) was given. On day 9, a second injection of 10 μg of GnRH was administered. The eCG-treated group also received an additional injection of 400 IU of eCG (Folligon^®^, Intervet Limited, Bangkok, Thailand) on day 4, while the untreated control group received an equivalent volume of PBS (Figure 1).

### 2.3. Ultrasound Assessment of Follicular Development and Ovulation

Ultrasonic evaluation of the ovarian structures was conducted using a color Doppler ultrasound machine (SonoscapeTM, Model E2V, Shenhen, China) equipped with a 5.0 MHz linear-array transducer for both B-mode and Doppler imaging. Consistent settings for frequency, gain, and color patterns were carefully selected and maintained throughout this study. The ovarian structure in both the untreated control and eCG-treated groups was assessed from day 0 to ovulation. The TFG was measured daily using the internal calipers on the ultrasound machine. The terminal follicle (TF) was identified as the preovulatory follicle throughout the study period. Ovulation was defined by the disappearance of the TF. The terminal follicular growth rate (TFGR) from day 0 to day 4 was calculated using the following formula: Growth rate = (diameter of selected follicle on day 4—diameter on day 0)/number of days between day 0 and day 4. The TFGR from day 4 to ovulation was calculated using the same formula. The terminal follicular diameter (TFD) was measured prior to ovulation or before the disappearance of the TF.

### 2.4. Ultrasound Assessment of CL Diameter and Luteal Area

CL development was studied and assessed on days 5–6 (designated as the developing CL) and days 9–12 (designated as the mid-stage CL) post-ovulation [29]. The location and diameter of each CL were recorded on an ovarian map, and ultrasound images were saved for subsequent analysis. The CL diameter was measured using the internal calipers on the ultrasound machine. The luteal area of the CL was calculated using the formula luteal area = π/4 × (mean CL diameter)^2^, where π = 3.1416 and the mean CL diameter was used. In cases where a cavity was present, the luteal area was adjusted by subtracting the area of the cavity, calculated as luteal area = π/4 × (mean CL diameter)^2^ − π/4 × (mean CL cavity)^2^. The CL diameter was measured in millimeters (mm), while the luteal area was expressed in square centimeters (cm^2^), respectively. All of the measurements were performed by the same technician to ensure consistency throughout this study.

### 2.5. Analysis of Tissue and Colored Areas of the CL

To assess the tissue and colored areas of the CL, Doppler color images were analyzed using Adobe Photoshop CC (version 2020). A series of still images and short video clips (<15 s) was captured to represent the maximum Doppler color signals of the CL. The tissue area of the CL was measured using the magnetic Lasso tool in Adobe Photoshop to outline the perimeter of the CL in the ultrasound images. This tool enabled the technician to define the tissue area in pixels. After outlining the CL, the total number of pixels corresponding to the tissue area was recorded. Similarly, the colored area within the CL, which indicates blood perfusion, was identified using the same magnetic Lasso tool. The areas showing color were delineated, and the number of pixels in these colored regions was quantified. To calculate the ratio of the colored area to the total tissue area of the CL, the percentage of the colored area relative to the tissue area was determined using the following formula: Proportion of colored area to CL tissue area (%) = (colored area (pixels)/tissue area (pixels)) × 100. All of the measurements were performed by the same technician to ensure consistency throughout this study. The analysis was based on the pixel density data for each image, with the results expressed as the colored area in pixels and the proportion of the colored area to the tissue area given as a percentage.

### 2.6. Assessment of Plasma P4 Levels

Plasma samples were collected from the jugular vein into heparinized tubes on the same days as the ultrasound examinations were conducted to monitor CL development. After their collection, the blood samples were promptly centrifuged at 4 °C at 800× *g* for 15 min to separate the plasma, which was stored at –20 °C until further analysis. P4 levels were quantified using competitive enzyme-linked immunosorbent assay (ELISA) following the protocol of the National Center for Genetic Engineering and Biotechnology, Thailand. The sensitivity for P4 using the ELISA assay was determined to be 1.4 ± 0.1 ng/mL, with intra-assay and inter-assay coefficients of variation of 9.8% and 14.2%, respectively.

### 2.7. Statistical Analysis

Continuous data are expressed as the mean ± standard error of the mean (SEM), while non-continuous data are presented as percentages. Data visualization was performed using scatter plots generated using GraphPad Prism version 8.0.2. The data were analyzed with an ANOVA using the general linear model (GLM) procedure. To compare the effect of the means of follicular growth and CL development between the untreated control and eCG-treated groups throughout the experiment, Student’s *t*-test was used. The proportion of CL color relative to CL area was analyzed using a Chi-square analysis. All of the statistical analyses were conducted using SAS 9.4 software (SAS Institute, Cary, NC, USA). Additionally, Pearson’s linear correlation coefficient was calculated to assess the strength of the relationships between various parameters, including follicular growth, luteal area, CL blood perfusion, and plasma P4 concentrations. The strength of the correlations was classified as weak (*r* ≤ 0.35), moderate (*r* = 0.36–0.67), or strong (*r* = 0.68–1.00). A significance level of *p* ≤ 0.05 was considered statistically significant. Pearson’s correlation results are presented as correlation coefficients (*r*), with the significance of each correlation tested using a two-tailed approach.

## 3. Results

### 3.1. Development and the TFGR

**Figure 2:**
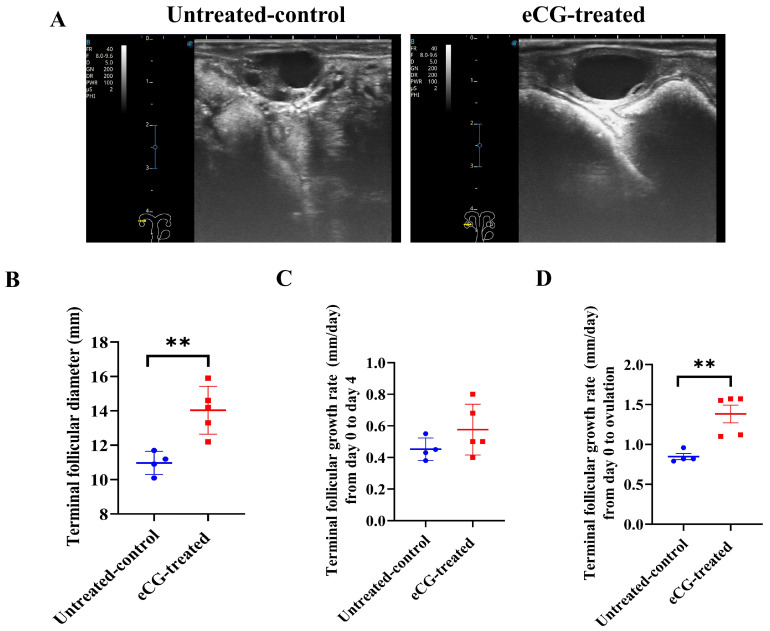
Development and growth rates of follicles in untreated control and eCG-treated groups. (**A**) Representative images of TF in both untreated control and eCG-treated groups prior to ovulation. (**B**) Terminal follicular diameter (mm) from day 0 to the day before ovulation. (**C**) Terminal follicular growth rate (mm/day) from day 0 to day 4. (**D**) Terminal follicular growth rate (mm/day) from day 0 to ovulation. Data are expressed as mean ± SEM. Statistical significance is indicated by asterisks (** *p* < 0.01). Data from *n* = 4 and *n* = 5 were included in the analysis, as the remaining 2 cows in the untreated control group and 1 cow in the eCG-treated group did not ovulate and were therefore excluded from the calculation.

Figure 2A presents representative images of the TF in both the untreated control and eCG-treated groups prior to ovulation. These images highlight the clear visual differences in TFG between the two groups, demonstrating the effect of the eCG treatment on follicular growth. The TF parameters were measured from day 0 to the day before ovulation. The results indicated a faster increase in the TFD in the eCG-treated group compared to that in the untreated control group. Statistically significant differences were observed, with the eCG-treated group exhibiting a larger TFD (*p* < 0.01) compared to that in the untreated control group (14.04 ± 0.62 vs. 10.98 ± 0.34 mm; Figure 2B). No significant difference (*p* > 0.05) was found in the TFGR between day 0 and day 4 in either group (Figure 2C). However, from day 4 to ovulation, the eCG-treated group exhibited significantly greater growth rates (*p* < 0.01) compared to those in the untreated control group (1.38 ± 0.11 vs. 0.85 ± 0.04 mm/day; Figure 2D). This suggests that the eCG treatment enhanced TFG, particularly in terms of the growth rates and the overall TFD before ovulation, compared to that in the untreated control group, which involved only the injection of PBS.

### 3.2. Development of the CL

**Figure 3:**
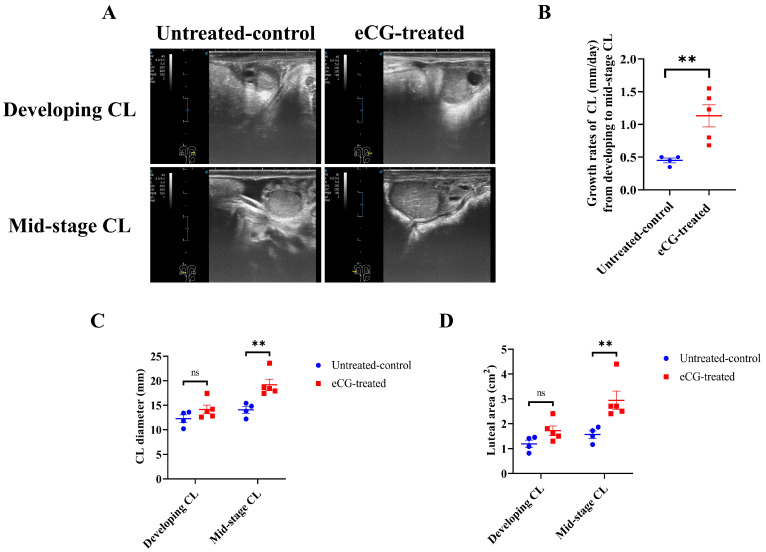
Development of CL in untreated control and eCG-treated groups. (**A**) Representative images of the developing and mid-stage CL in both untreated control and eCG-treated groups. (**B**) Growth rates of the CL (mm/day) from the developing CL on days 5–6 to the mid-stage CL on days 9–12 post-ovulation. (**C**) CL diameter (mm) at different stages of development. (**D**) Luteal area (cm^2^) measured for the developing and mid-stage CL. Data are presented as means ± SEMs. Statistical significance is indicated by asterisks (** *p* < 0.01), and non-significant differences are denoted by “ns”. Data from *n* = 4 and *n* = 5 were included in the analysis, as the remaining 2 cows in the untreated control group and 1 cow in the eCG-treated group did not ovulate and were therefore excluded from the calculation.

Figure 3A presents representative images of developing and mid-stage CLs from both the untreated control and eCG-treated groups. These images clearly illustrate the differences in the appearance and progression of the CL between the two groups, particularly at various stages post-ovulation. The growth rates of the CL, from the developing to the mid-stage CL, were significantly higher (*p* < 0.01) in the eCG-treated group, indicating accelerated CL development compared to that in the untreated control group (1.13 ± 0.17 vs. 0.45 ± 0.04 mm/day; Figure 3B). There were no significant differences (*p* > 0.05) in the CL diameter or the luteal area at the developing CL stage between the two groups (Figure 3C,D). However, for the mid-stage CL, the eCG-treated group showed significantly larger values (*p* < 0.01) in terms of CL diameter (19.18 ± 1.12 vs. 14.05 ± 0.70 mm; Figure 3C) and luteal area (2.93 ± 0.37 vs. 1.56 ± 0.15 cm^2^; Figure 3D). These findings suggest that eCG treatment enhances CL growth, with significant increases in its diameter and area. Taken together, these results indicate that eCG treatment accelerates CL development and maturation, promoting more substantial luteal tissue growth compared to that in the untreated control group. This underscores the effectiveness of eCG in enhancing both follicular and luteal development.

### 3.3. Blood Perfusion Indices and Plasma P4 Levels

**Figure 4:**
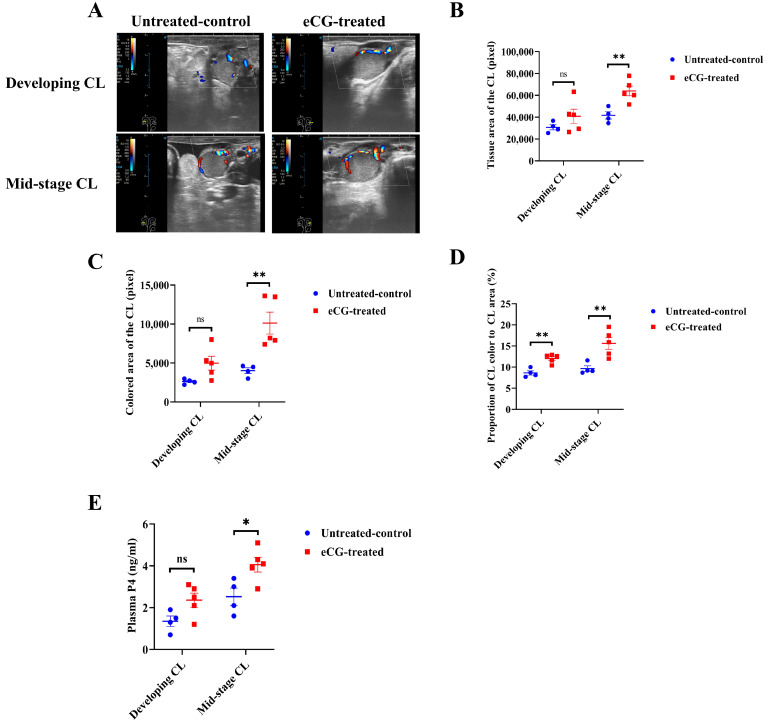
Blood perfusion indices and plasma P4 levels in developing and mid-stage CLs in untreated control and eCG-treated groups. (**A**) Representative images illustrating blood perfusion in the developing and mid-stage CLs in both the untreated control and eCG-treated groups. (**B**) Tissue area of the CL (in pixel) at different stages of development. (**C**) Colored area of the CL (in pixel), reflecting blood perfusion, at different stages of development. (**D**) Proportion of the colored area to the whole tissue area of the CL (%) at different stages of development. (**E**) Plasma P4 concentrations (ng/mL) during CL development. Data are presented as means ± SEMs. Statistical significance is indicated by asterisks (** *p* < 0.01 and * *p* < 0.05), and non-significant differences are denoted by “ns”. Data from *n* = 4 and *n* = 5 were included in the analysis, as the remaining 2 cows in the untreated control group and 1 cow in the eCG-treated group did not ovulate and were therefore excluded from the calculation.

Figure 4A illustrates representative images of the blood perfusion in the developing and mid-stage CLs in both the untreated control and eCG-treated groups. These images highlight notable differences in the blood perfusion between the two groups at various stages of CL development. The eCG-treated group exhibited more pronounced blood perfusion in both the developing and mid-stage CLs compared to that in the untreated control group. No significant differences (*p* > 0.05) were observed in the tissue or colored areas between the two groups at the developing CL stage. However, for the mid-stage CL, the eCG-treated group displayed significantly larger tissue (*p* < 0.01) (64,072.4 ± 4420.9 vs. 41,721.0 ± 3328.5 pixel; Figure 4B) and colored (10,128.0 ± 14.05.3 vs. 4013.3 ± 366.8 pixel; Figure 4C) areas than these values in the untreated control group. The proportion of the colored area to the entire tissue area in the CL was also significantly higher (*p* < 0.01) in the eCG-treated group for both the developing CL (12.0 ± 0.5 vs. 8.6 ± 0.5%; Figure 4D) and the mid-stage CL (15.26 ± 1.4 vs. 9.6 ± 0.7%; Figure 4D). These findings suggest that eCG treatment enhances the blood perfusion relative to the total tissue area of the CL at both stages of development. In terms of the plasma P4 concentrations during CL development, no significant differences (*p* > 0.05) were observed between the two groups at the developing CL stage. However, for the mid-stage CL, the eCG-treated group exhibited significantly higher plasma P4 levels (*p* < 0.05) compared to those in the untreated control group (4.1 ± 0.3 vs. 2.5 ± 0.4 ng/mL; Figure 4E). This indicates that eCG treatment not only enhances CL blood perfusion but also promotes P4 production during the luteal phase. These results collectively demonstrate that eCG treatment enhances the blood perfusion and plasma P4 levels during CL development. Specifically, the increased blood perfusion and elevated plasma P4 concentrations observed in the eCG-treated group suggest that eCG plays a crucial role in facilitating CL development and function. Enhanced blood perfusion may contribute to more efficient hormone production, further supporting the role of eCG in promoting both follicular and luteal growth.

### 3.4. Pearson’s Correlation Coefficients for Various Parameters of the TF, the CL, and P4 Concentration

**Figure 5:**
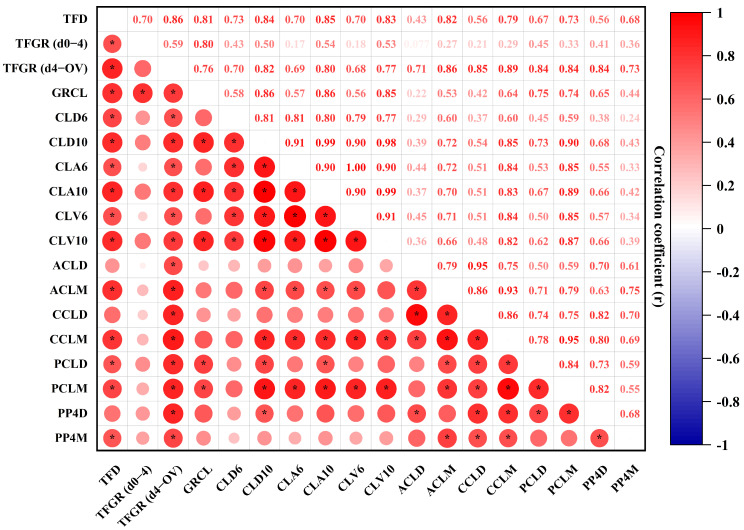
Pearson’s correlation coefficients (*r*) for various parameters of the TF, CL, and P4 concentration. Statistical significance was considered at a *p*-value of ≤0.05, as indicated by an asterisk. Abbreviations: TFD (terminal follicular diameter); TFGR (d0–4) (terminal follicular growth rate from day 0 to day 4); TFGR (d4–OV) (terminal follicular growth rate from day 4 to ovulation); GRCL (growth rate of the CL for the developing to the mid-stage CL); CLD6 (diameter of the developing CL); CLD10 (diameter of the mid-stage CL); CLA6 (the CSA of the developing CL); CLA10 (the CSA of the mid-stage CL); CLV6 (the volume of the developing CL); CLV10 (the volume of the mid-stage CL); ACLD (the tissue area of the CL for the developing CL); ACLM (the tissue area of the CL for the mid-stage CL); CCLD (the colored area of the CL for the developing CL); CCLM (the colored area of the CL for the mid-stage CL); PCLD (the proportion of CL color to the CL area for the developing CL); PCLM (the proportion of CL color to the CL area for the mid-stage CL); PP4D (plasma P4 of the developing CL); PP4M (plasma P4 of the mid-stage CL). Statistical significance is denoted by asterisks for *p* < 0.05.

The Pearson’s correlation coefficients were calculated to evaluate the relationships between various parameters of the TF, the CL, and P4 concentration (Figure 5). The results are presented as Pearson’s correlation coefficients (*r*), with varying color intensities indicating the strength of the correlations. As illustrated in the correlation map, several parameters associated with the TF, CL, and P4 concentration exhibited significant correlations. For example, the TFGR from day 4 to ovulation showed strong positive correlations (*r* = 0.68–1.00) with the CL’s diameter, CSA, and volume for both developing and mid-stage CLs, suggesting that these parameters are closely associated with TF and CL development. Similarly, the proportion of the colored area to the entire tissue area of the CL demonstrated a strong positive correlation with PP4D, while moderate to strong correlations (*r* = 0.36–0.67) were observed with PP4M, indicating that increased blood perfusion and CL maturation are closely linked to elevated P4 production. Furthermore, weak correlations (*r* ≤ 0.35) were found between the TFGR from day 0 to 4 (d0–4) and various CL parameters, such as the CLA6, CLV6, ACLD, ACLM, CCLD, CCLM, and RCLM. These findings suggest a potential relationship between early TFG and the subsequent stages of CL maturation. These results highlight the complex interrelationships between TFG, CL development, and P4 production. The varying strengths of the correlations offer valuable insights into the physiological process underlying follicular and luteal maturation.

## 4. Discussion

The main findings of this study indicate that the administration of eCG significantly enhanced the TFG and luteal development in White Lamphun cows. Our Doppler ultrasound observations revealed that the eCG-treated group exhibited superior CL blood perfusion compared to that in the untreated control group following ovulation. Additionally, the eCG-treated group demonstrated accelerated TFG and a larger TFD relative to these values in the untreated control group. This enhanced growth was associated with an increased CL size and elevated plasma P4 concentrations, particularly during the development and mid-stage phases for the CL. These findings emphasize the beneficial effects of eCG treatment on both follicular and luteal dynamics, suggesting that eCG not only stimulates follicular development but also supports optimal luteal function. Consequently, eCG administration may contribute to improved reproductive performance and fertility outcomes in White Lamphun cows.

In general, eCG administration enhanced the TFG and TFD, consistent with previous studies that reported a significantly higher TFGR in the eCG-treated group (1.45 mm/day) compared to that in the control group (0.90 mm/day) [30]. Notably, eCG maintains active concentrations in the bloodstream for up to three days [18], which may contribute to its ability to effectively stimulate follicular growth. The extended half-life of eCG is likely one of the key reasons for its success in promoting follicular development, as observed in the present trial. Similar findings have been reported in previous research [31], where heifers and cows treated with eCG experienced doubled follicular growth rates (eCG = 1.2 to 1.5 mm/day compared to the control = 0.5 to 0.6 mm/day). These results further corroborate the notion that eCG plays a crucial role in enhancing follicular growth through various mechanisms. The binding of LH to the granulosa cell receptors initiates a cascade of reactions that stimulate the production of catalytic enzymes responsible for steroidogenesis, which in turn promotes follicular growth [32]. It is believed that eCG induces a similar cascade of reactions, as it has been shown to enhance estradiol production by upregulating the mRNA for P45017α [33]. Consequently, eCG appears to act as a gonadotropic agent, functioning similarly to LH to stimulate TFG [34]. This mechanism is particularly effective in cows with a compromised ovarian function or poor LH release into the bloodstream, as described in previous studies [14,31]. The scientific literature highlights the close correlation between the diameter of the ovulated TF and subsequent CL development during the diestrus phase [35]. Furthermore, CL development is strongly linked to circulating P4 concentrations [36]. Thus, it is plausible that eCG-induced ovulation from a larger TF leads to the formation of a larger CL, resulting in increased circulating P4 levels.

The TF typically forms a functional CL when it reaches a diameter of 10.5–12.0 mm [37], and cows with larger TFs and CLs tend to exhibit higher ovulation rates, pregnancy rates, and P4 levels compared to those with smaller structures [38,39]. The CL is a transient endocrine gland that forms after ovulation and is primarily responsible for producing P4, a hormone critical for the establishment and maintenance of pregnancy [40]. Previous studies have shown that the diameter of the ovulated TF is strongly correlated with the amount of blood perfusion in the follicular wall, and selecting an ovulated TF with greater blood perfusion may enhance the plasma P4 production by the CL in beef cattle [41]. Notably, treatment with eCG has been shown to increase the blood perfusion and volume density of the CL and stimulate angiogenesis, thus promoting CL function [42]. Previous studies have indicated that a higher percentage of eCG-treated cows became pregnant following TAI, particularly when eCG was included in the TAI protocol [30]. These findings highlight the positive impact of eCG not only on enhancing follicular growth and ovulation but also on stimulating the formation of and improving the development of the CL, which, in turn, creates a uterine environment that is more favorable for embryonic development [11]. For instance, the administration of eCG on day 5 of a synchronization protocol has been shown to result in a larger CL by day 9 after ovulation, with a corresponding increase in P4 concentrations. Furthermore, in anestrous cows treated with eCG 2 days before the removal of a P4-releasing device during an 8-day FTAI protocol, both the CL volume and P4 concentrations were greater at 10 and 15 days post-ovulation [43]. Our data align with the concept that the P4 concentrations plateau at the mid-stage of CL development post-ovulation [44], with a strong correlation between CL size and luteal function during CL development [45]. Additionally, cows having higher P4 concentrations shortly after ovulation is linked to improved fertility outcomes [46]. The FSH- and LH-like activity of eCG, which results in the formation of a larger TF, likely increases the number of granulosa and theca cells, indirectly boosting the number of large luteal cells responsible for P4 production during diestrus [47]. Research has shown a positive correlation between CL size and P4 production in cows, with a larger CL associated with enhanced blood perfusion and increased P4 synthesis in pregnant dairy cows [48,49]. Furthermore, cows with larger TFs tend to have greater follicle blood perfusion, which can influence subsequent CL development [41]. Similar findings have been observed in mares, where the TFD positively affects the CL diameter, blood perfusion, and P4 production [50]. Likewise, in beef cows, a larger TF results in higher P4 concentrations and pregnancy rates in cattle [45,51], and ultrasonographic measurements of the CL area can accurately predict P4 levels, with a CL area of 3.3 cm^2^ or greater indicating a functional CL in dairy cows [52].

These findings suggest that the TFD can serve as a reliable indicator of the subsequent luteal function and fertility potential in bovine species. While the effects of eCG on TF and CL development were evident, the extent of its enhancement may vary depending on factors such as the administration timing, the dosage, and the reproductive status of the animals. Further studies are needed to optimize the eCG protocols for White Lamphun cattle and investigate its long-term effects on their reproductive performance. Larger sample sizes and the use of FTAI should be included in future investigations to confirm these results. In conclusion, eCG treatment shows promise in enhancing TF and CL development in White Lamphun cattle, offering insights into fertility management through hormonal interventions. Future research should refine the eCG protocols and explore their application in breeding programs to enhance productivity and reproductive efficiency.

## 5. Conclusions

This study clearly demonstrates that the addition of eCG to the estrous synchronization protocol accelerates TFG, leading to a larger TFD and higher growth rates, particularly between days 4 and ovulation. The accelerated TFG and greater TFD, accompanied by enhanced growth rates, further stimulated faster CL development, resulting in a significantly increased CL size and luteal area at the mid-stage. Additionally, cows exhibiting higher TFG, TFDs, and growth rates showed increased blood perfusion and elevated plasma P4 levels during both the developing and mid-stage CL phases. This suggests that eCG administration improves follicular development, which plays a crucial role in optimizing luteal development and function. The Pearson’s correlation analysis revealed a strong relationship between follicular development, CL development, and P4 production, highlighting their critical role in fertility. Overall, these findings emphasize the positive impact of eCG supplementation in estrous synchronization protocols, improving reproductive performance by promoting both follicular and luteal maturation.

## Data Availability

The data that support the findings of this experiment are available from the corresponding authors upon reasonable request.
